# Non–adherence and predictors in patients with schizophrenia on second generation antipsychotics at Amanuel Mental Specialized Hospital, Ethiopia

**DOI:** 10.1371/journal.pone.0314403

**Published:** 2025-03-26

**Authors:** Melak Gedamu Beyene, Solomon Teferra, Teferi Gedif Fenta

**Affiliations:** 1 School of Pharmacy, College of Health Sciences, Addis Ababa University, Addis Ababa, Ethiopia; 2 Department of Psychiatry, School of Medicine, College of Health Sciences, Addis Ababa University, Addis Ababa, Ethiopia; University of Toronto, CANADA

## Abstract

**Background:**

Schizophrenia is a chronic and profound mental disorder. Non–adherence to prescribed medication regimens is a major concern in the treatment of schizophrenia. This study aimed to investigate the prevalence, factors, and reasons contributing to non–adherence among Ethiopian patients with schizophrenia who are receiving second-generation antipsychotics (SGAs).

**Methods:**

A hospital–based cross–sectional study was done at Amanuel Mental Specialized Hospital (AMSH) from 03/10/2022 to 31/8/2023. Data were collected using the drug attitude inventory-10 (DAI-10) tool. Analysis was conducted using Statistical Package for Social Sciences (SPSS) version 25. Univariate and multivariate binary logistic regression analyses were conducted.

**Results:**

Most participants were male, (90.0%), and aged 26-40 years, (53.5%). Mean doses for risperidone and olanzapine prescribed were 4.9mg (±2.4) and 13.5mg (±5.0), respectively. Close to 40% of patients were khat (Catha edulis) users. The mean Positive and Negative Syndrome Scale (PANSS) total score was 71.1 ± 35.9 and the Clinical Global Impression-Severity (CGI-S) score was 3.42 ± 1.21. Around 31.4% of participants were non-adherent. Forgetfulness (31.73%) and stigma (27.31%) were the primary reasons cited for non–adherence. The multivariate binary logistic regression analysis revealed a significant association between non–adherence and several key factors. Patients with a PANS score equal to or greater than 71 (95% CI: 1.04, 3.80; p = 0.02), Patients categorized as having a moderate to markedly severe illness (95% CI: 1.1, 3.1; p = 0.024), duration of follow-up (DUP) (6-10) years (95% CI: 1.4, 129; p = 0.02), and age (26-40) years (95% CI: 0.3, 0.9; p = 0.04) were found to be statistically significant predictors of non–adherence.

**Conclusion:**

The investigators recommended that counseling of the patients to highlight the importance of adherence, instituting regular and comprehensive symptom monitoring, tailoring interventions to address reasons for non-adherence, promoting early intervention and treatment initiation to reduce the DUP, and customizing interventions based on age-specific needs.

## 1. Introduction

Schizophrenia is a persistent and severe mental disorder [1]. Although many people experience symptom improvements at first, most will experience more episodes of schizophrenia [2]. Typical disease stages include repeated phases of worsening and improving symptoms, and some patients are unable to recover completely from these episodes, resulting in health problems that accumulate. Consequently, there is a potential for progressive functional decline [3].

The introduction of antipsychotic medications has enhanced the well-being of individuals with schizophrenia by effectively managing their symptoms [4,5]. The review by Fabrazzo et al., underscores the superior effectiveness and tolerability of second-generation antipsychotics (SGAs) in real-world settings for treating schizophrenia and related disorders. It highlights important considerations for clinical practice, such as the need for improved treatments targeting negative and cognitive symptoms and the potential benefits of long acting injectables in enhancing adherence and clinical outcomes [5]. Similarly, other studies also highlighted the advantages of SGAs over first-generation antipsychotics (FGAs) in terms of effectiveness, tolerability, and social rehabilitation benefits, adherence and continuation of treatment [6,7]. However, non-adherence to long-term medication therapy is a significant concern, especially in schizophrenia, where continuous medication is essential, and it results in substantial costs [8,9]. Over a 12-month treatment period, adherence rates to SGAs are reported to range from approximately 50% to around 60% [10,11]. Not-adhering to the prescribed treatment is linked to elevated rates of symptom reappearance, an increased risk of relapse, and a greater probability of needing emergency room visits and hospitalization [12]. In a research study carried out in Ethiopia to assess non-adherence rates of antipsychotic medications in patients diagnosed with schizophrenia, the findings indicated a variation from 19.6% - 50.2% [13–17].

One systematic review reported identified modifiable risk factors for non-adherence which was categorized into patient-related factors and environment [18].These findings align with Clinical Antipsychotic Trials of Intervention Effectiveness, which found that 75% of patients discontinued phase I antipsychotic medication within 18 months, often due to personal decisions rather than efficacy or side effects [19].

Another study found that the most influential factor promoting medication adherence was the perceived daily advantages of the treatment. This was closely followed by positive family support, relapse prevention, positive outlook towards treatment, and family-related pressures or coercions. In contrast, the most common cause of non-adherence was the denial of illness. Additional notable factors contributing to non-compliance included financial constraints, limited awareness of the illness, reduced accessibility to treatment facilities, medication side effects, and substance abuse [16,20–22].

Qualitative research study conducted in Ethiopia identified key factors in non-adherence, including limited access to food to counteract medication-induced appetite issues, perceptions of drug potency, and the absence of a social safety net. Patients’ expectations of complete recovery and issues like lack of insight, treatment response, mental health stigma, and dissatisfaction with healthcare providers were significant contributors to non-adherence, mirroring challenges in wealthier nations [23]. Another study conducted in Ethiopia reported forgetfulness, being busy, lower levels of literacy, and belonging to an older age group, searching for alternative therapies, unavailability of medications, and lack of sufficient information as one of the main reasons for non-adherence to the drug [14–16].

A high pill burden, extended maintenance therapy, residing in rural areas and current substance use (particularly in cases of long treatment duration and polypharmacy) were also significant predictors of non-adherence. Moreover, patients who were compelled to take medication against their will, those who did not perceive a need for medication, and those who discontinued their medication without consulting their prescriber were also significantly associated with non-adherence [13,17].

In the context of Ethiopia, research study on medication non–adherence, particularly regarding SGAs, has seen some notable gaps. While studies have been conducted in Ethiopia to investigate adherence and the factors, and reasons contributing to non-adherence among schizophrenia patients on different antipsychotics, the present study focus on patients who were using SGAs. These medications are known for its unique side effects profile predisposing to non-adherence [12,24].

In addition, this study was conducted in a less researched country and addresses a significant gap in the literature. By highlighting the unique sociodemographic and cultural factors influencing non-adherence in Ethiopian context, our research can offer insights that are not covered in previous studies.

This gap is significant because schizophrenia often requires long-term, consistent medication regimens, making adherence a crucial factor in the management and well-being of affected individuals. SGAs, which are commonly used in the treatment of these conditions, come with unique challenges and considerations, including metabolic side effects and variability in long-term effectiveness [25,26].

Understanding the specific factors that influence adherence to SGAs in the Ethiopian context is crucial for healthcare providers and policymakers. Such knowledge can inform the development of targeted interventions and support mechanisms to enhance treatment adherence and, subsequently, improve the overall health and quality of life of individuals with schizophrenia in Ethiopia. Therefore, bridging this gap in research is essential to address the distinct challenges associated with SGAs use.

## 2. Materials and methods

### 2.1. Study settings

This research was carried out at Amanuel Mental Specialized Hospital (AMSH) situated in Addis Ababa, Ethiopia. Founded in 1930, AMSH is serving as the sole mental health facility in the nation. The hospital provides outpatient services, with approximately 115,000 outpatient visits. AMSH also fulfills the role of a referral hospital for psychiatry cases from all regions of the country [27].

### 2.2. Study design and period

A hospital-based cross-sectional study was conducted at AMSH from 03/10/2022 to 31/08/2023.

### 2.3. Source and study population

The source population consisted of all patients attending AMSH who were participants in the Neuropsychiatric Genetics in African Populations (NeuroGAP-psychosis) project [28]. The study population consisted of all patients diagnosed with schizophrenia who had been administered either risperidone or olanzapine and met the inclusion criteria within the study timeframe.

### 2.4. Eligibility criteria

#### 2.4.1. Inclusion criteria.

The study participants included were those who had no physical complications or other psychiatric disorders, lacked a history of resistance to antipsychotic treatment, met the Diagnostic and Statistical Manual of Mental Disorders (DSM-5) [29] criteria for schizophrenia, were on risperidone or olanzapine monotherapy for at least three months, and whose concomitant medication use was restricted to benzodiazepines for anxiety or agitation and anticholinergics, age greater or equal to 18 years, and voluntary to participate in the study.

#### 2.4.2. Exclusion criteria.

Those patients in acute psychotic episode, diagnosis of severe, unstable medical or neurological conditions, presence of mental retardation and severe cognitive deterioration that would make it difficult for the patient to understand the questions were excluded in the study.

### 2.5. Study variables

#### 2.5.1. Dependent variables.

The treatment non-adherence status of patients with schizophrenia, which was assessed by drug attitude inventory-10 (DAI-10) [30] rating scale and classified as adherent or non–adherent was the dependent variable.

#### 2.5.2. Independent variables.

The independent variables were socio-demographic characteristics such as age, gender, marital status and income level, clinical characteristics (medications prescribed, comorbidity, duration of the disease, time of treatment initiation, dose of the drug, duration of treatment, Clinical Global Impressions-Severity (CGI-S) score, Positive and negative syndrome scale (PANSS) score and social drug use history.

### 2.6. Data collection tools

#### 2.6.1. Medication adherence assessment tool.

To measure medication adherence, the researchers employed a tool called the DAI-10. This scale was based on the original 30-item scale. DAI Scores are allocated to each answer and the total score is calculated in the same way as for the DAI-30 [30,31]. Items 1, 3, 4, 7, 9, and 10 reflect favorable attitudes towards the current medication, while items 2, 5, 6, and 8 convey negative attitudes. Positive affirmations are scored as + 1, and negative ones as -1. The final score is the sum of the positives and negatives. The DAI-10 scoring ranges from -10 to + 10. Patients who receive a total score greater than zero are classified as adherent, whereas those with a score equal to or less than zero are categorized as non–adherent to their medication with Cronbach’s alpha of 0.81 [32–35].

The data collection process involved translating the DAI-10 questionnaire into Amharic, followed by a back-translation into English, which two separate translators carried out.

#### 2.6.2. Clinical data collection tool.

PANSS was used to measure the symptom severity of the disease. It is a clinical instrument widely used in the field of psychiatry to assess the severity of symptoms in schizophrenia patients. PANSS is a structured interview-based assessment system that provides a comprehensive assessment of positive symptoms (e.g., hallucinations and delusions), negative symptoms (including social withdrawal and lack of motivation) and general psychological diseases (covering different symptoms that are not specific to positive or negative categories). The scale consists of 30 items ranked on a seven-point scale, and the highest scores show a greater symptom severity. The original standardization of the PANSS-30 has demonstrated good internal consistency, although the exact value was not provided. Subsequent research has consistently supported the reliability of the PANSS-30 with Cronbach’s alpha values generally ranging from 0.70 to 0.90 [36,37]. PANSS is valuable for clinicians and researchers to quantify symptom severity, track changes over time, and assess the efficacy of interventions in individuals with schizophrenia or related psychotic disorders [38].

CGI-S was used to measure the severity of the disease. Widely employed in psychiatric research, this tool offers a comprehensive assessment of illness severity by capturing the clinician’s holistic impression of the patient’s condition. As an integral component of the broader CGI scale, the CGI-S entails clinicians assigning a severity rating on a seven-point scale, ranging from “1 = normal, not at all sick” to “7 = among the sickest.” CGI-S plays a crucial role in evaluating treatment effectiveness and overarching clinical improvement [39]. The CGI-S scale, while typically a single-item measure and less commonly analyzed using Cronbach’s alpha, has shown significant overlap and concurrent validity with the PANSS. This correlation further supports the reliability of the CGI-S as a measure of symptom severity in schizophrenia [36,40].

In order to methodically extract clinical data from the patient’s medical charts, a data abstraction format was created. The provided medications, their dosages, and any pertinent clinical information that would have an influence on their adherence to the treatment plan were all gathered using this format.

#### 2.6.3. Questionnaires for sociodemographic data.

Questionnaires were prepared by researchers to gather sociodemographic information from study participants in order to collect information regarding the patient’s background, including age, gender, occupation, education, and other sociodemographic factors that may have an impact on adherence.

#### 2.6.4. Questionnaires for reasons for non-adherence.

Additionally, questionnaires were designed to capture information on the reasons for non-adherence. These questionnaires allowed the researchers to understand the specific factors or circumstances that led patients to not adhere to their prescribed medication regimens.

### 2.7. Sample size and data collection procedures

A total of 271 patients were included in the study and the participants were recruited from 03/10/2022 to 31/08/2023. The data administration office of the NeuroGAP project made available a database that included detailed information on individuals with schizophrenia diagnoses who are presently receiving treatment with olanzapine or risperidone. These patients’ identifying numbers are obtained, and their phone numbers and other contact information are then collected to facilitate direct communication. Medical charts of patients who agreed to take part in the study were obtained from the hospital card office. Patients were contacted using their phone numbers they provide to facilitate patient engagement. Upon first contact, the research objectives were explained to the participants. Patients willing to participate were invited to come to the hospital for interview, and their medical charts were retrieved.

### 2.8. Data quality assurance

Data quality assurance was a paramount concern throughout the study. Several measures were implemented to uphold data accuracy and integrity. A pretest was conducted on 5% of the sample size at Zewditu Memorial Hospital to identify potential issues or ambiguities in the data collection. Moreover, the clarity and comprehensibility of the questionnaire were closely scrutinized, and necessary amendments were made to enhance its precision.

Comprehensive training sessions were organized to ensure data collectors were well-prepared, equipping them with the skills required for accurate data collection. Continuous supervision by the principal investigator was maintained to address potential challenges and maintain data quality.

The collected data was meticulously checked for completeness to mitigate gaps or missing information. A double-entry approach was employed in Statistical Package for Social Science (SPSS), followed by a thorough verification process. These steps were crucial to eliminate errors and inconsistencies and to guarantee data accuracy for analysis and interpretation.

### 2.9. Data processing and analysis

The data underwent a rigorous processing phase to ensure its reliability and quality. This included thorough cleaning to resolve incompleteness issues, then coding, input, and analysis using SPSS version 25. For categorical data, descriptive statistics were generated and presented as frequency and percentage. Mean, standard deviation (SD), and range were computed for continuous variables. Both univariate and multivariate binary logistic regression analyses were done to identify predictors of non–adherence.

A p-value of less than 0.25 from the univariate analysis was used to select candidate variables for the multivariable binary logistic regression analysis. Following adjustment from univariate logistic regression analyses, PANSS total score, CGI-S score, DUP, religion, age, alcohol use history and employment status were considered candidates for inclusion in the multivariable logistic regression model. A p-value less than 0.05 were deemed significantly associated with the outcome variable. Adjusted odds ratios (AOR) and 95% confidence intervals were used to report the association between independent variables and the outcome variable.

### 2.10. Ethics considerations

Ethical clearance was obtained from Ethical Review Committee of Amanuel Mental Specialized Hospital (Approval Number: AM/140/2/48) and the Institutional Review Board (IRB) of Addis Ababa University, College of Health Sciences (protocol number: 065/21/SoP). Written consent was obtained from all study participants. We ensured that participants were fully informed about the study’s objectives. To safeguard the confidentiality and privacy of the participants, data was analyzed in aggregates without including the patient identifiers. Non–adherent patients were given counselling on the importance of medication adherence.

## 3. Results

The binary logistic regression model fitness test was done using the Hosmer and Lemeshow test. The binary logistic regression model fits the data well, as evidenced by the non-significant p-value that was obtained, which suggests that there is no significant difference between the predicted and observed values.

### 3.1. Sociodemographic characteristics

The mean age of the study population was 34.2 with SD of 10.5 years and ranged from 18–85 years. Most of the study participants were male, 244 (90.0%), and aged 26-40 years, 145 (53.5%). Educational background shows variations in literacy levels, with significant proportions having completed secondary education, 124 (45.8%). Place of residence indicates a predominant urban population, 213 (78.6%). The majority of them were single, 173 (63.8%). Employment status depicts that most participants were unemployed, 153 (56.5%). Living arrangements are primarily with relatives,248 (91.5%), and almost half of the participants were Orthodox Christian, 128 (47.2%) (Table 1).

**Table 1:** Sociodemographic characteristics of patient with schizophrenia, Ethiopia (N = 271).

Variables	Frequency (N)	Percent (%)
Gender		
Male	244	90.0
Female	27	10.0
Age		
18-25	62	22.9
26-40	145	53.5
41-50	43	15.9
51-85	21	7.7
Educational background		
Illiterate/no formal education	7	2.6
Primary education	70	25.8
Secondary education	124	45.8
Tertiary education	70	25.8
Place of residence		
Urban	213	78.6
Rural	58	21.4
Marital status		
Single	173	63.8
Married	61	22.5
Divorced	37	13.7
Health service fee		
Free	18	6.6
Paying out-of-pocket	45	16.6
CBHI	208	76.8
Employment status		
Employed	118	43.5
Unemployed	153	56.5
Living arrangement		
Alone	22	8.1
Relatives	248	91.5
In-charity	1	0.4
Religion		
Orthodox Christian	128	47.2
Muslim	104	38.4
Protestant	34	12.6
Others *	5	1.8

Others * : catholic, no religion; CBHI: community-based health insurance

### 3.2. Clinical characteristics

This study’s findings revealed that most patients were prescribed with risperidone, 155 (57.2%). Regarding the equivalent dose of chlorpromazine (CPZ), a significant portion of patients received doses of less than 300 mg, 138 (50.9%). The mean dose of risperidone and olanzapine was 4.9mg with SD (±2.4) and 13.5mg with SD (±5.0), respectively. The daily dose range of risperidone and olanzapine was found to be 1-12 mg and 5-30 mg, respectively. Substance use was notably prevalent, with 108 (39.9%) of patients were khat (Catha edulis) users. The mean PANSS total score was 71.1 ± 35.9, the PANSS negative score was 17.2 ± 9.6, the PANSS positive score was 17.5 ± 9.7, the PANSS Psychopathology score was 36.4 ± 19.6, and the CGI-S score was 3.42 ± 1.21 (Table 2).

**Table 2:** Clinical characteristics of patients with schizophrenia, Ethiopia (N = 271).

Variables	Frequency (N)	Percent (%)
Type of SGA		
Risperidone	155	57.2
Olanzapine	116	42.8
CPZ equivalent dose		
<300 mg	138	50.9
300-600 mg	133	49.1
Substance use		
Khat		
Yes	108	39.9
No	163	60.1
Cigarette		
Yes	98	36.2
No	173	63.8
Alcohol		
Yes	77	28.4
No	194	71.6
Comorbidity		
Yes	13	4.8
No	258	95.2
Duration of illness		
<1 year	31	11.4
1-5 years	104	38.4
6-10 years	54	19.9
>10 years	82	30.3
DUP		
< 1 year	190	70.1
1-5 year	71	26.2
6-10 years	6	2.2
>10 years	4	1.5
Duration of treatment		
<1 year	37	13.7
1-5 years	115	42.4
6-10 years	50	18.4
>10 years	69	25.5

DUP: Duration of Untreated Psychosis; CPZ: chlorpromazine; SGA: Second Generation Antipsychotic

The mean total scale score for DAI-10 respondents in this study was 2.5 ± 3.3. The major findings from the provided table suggest that patients’ attitudes toward medication are diverse. The majority of respondents believe that the positive aspects of medication outweigh the negative ones (88.2%, Question 1), However, there is a notable portion who report feeling “doped up” on medication (55.4%, Question 2) and experiencing tiredness and sluggishness (62.4%, Question 5). Other details are highlighted below (Table 3).

**Table 3:** Frequency of patients’ response to drug attitude inventory item scale by patients with schizophrenia, Ethiopia (N = 271).

No.	Questions	Response	N	%
1	For me, the good things about medication outweigh the bad	True	239	88.2
2	I feel strange, ‘doped up’, on medication	True	150	55.4
3	I take medications of my own free choice	True	177	65.3
4	Medications make me feel more relaxed	True	193	71.2
5	Medication makes me feel tired and sluggish	True	169	62.4
6	I take medication only when I feel ill	True	162	59.8
7	I feel more normal on medication	True	201	74.2
8	It is unnatural for my mind and body to be controlled by medications	True	200	73.8
9	My thoughts are clearer on medication	True	230	84.9
10	Taking medication will prevent me from having a breakdown	True	246	90.8

### 3.3. Prevalence and reasons for non-adherence

This study determined the prevalence of non-adherence among patients with schizophrenia who were prescribed SGAs using the DAI-10 tool. The result revealed that about one-third of the study subjects, 85 (31.4%), were non-adherent to their medication.

**Fig 1:**
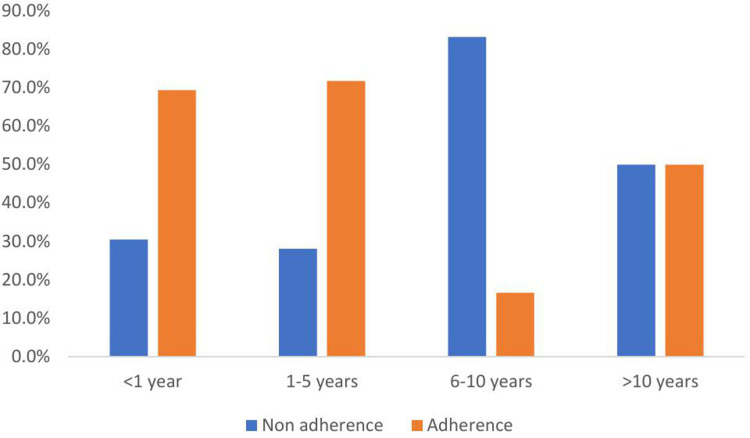
Adherence level and years of untreated psychosis among patients with schizophrenia, Ethiopia (N **=** **271).** The following figure showed that among patients classified as “borderline to mildly ill,” a majority are adherent, 117 (74.1%) (Fig 2) and (S2 Table).

The following figure depicts the potential influence of the DUP on medication non-adherence among schizophrenia patients. The non-adherence rate is higher in longer DUP (DUP of 6-10 years, 83.3%) (Fig 1) and (S1 Table).

**Fig 2:**
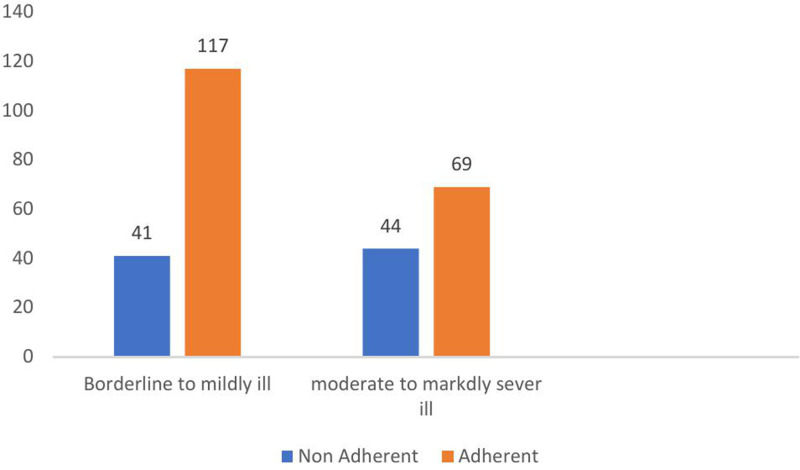
Adherence status with respect to CGI-S score among patients with schizophrenia, Ethiopia (N **=** **271).** Similarly, the following figure depicts that the majority of the study participants were non-adherent in patients with higher PANSS scores, 67 (24.7%) (Fig 3) and (S3 Table).

**Fig 3:**
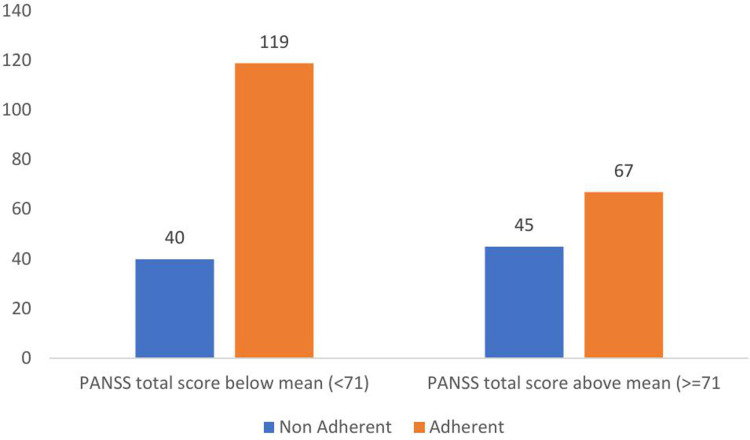
Adherence status with respect to PANSS score in patients with schizophrenia, Ethiopia (N **=** **271).** PANSS: Positive and Negative Syndrome Scale. Among the patients participating in the study, the research delved into the reasons for non-adherence to SGA treatment. Notably, forgetfulness emerged as the most commonly cited reason, affecting 86 (31.7%) of the study participants, followed by the presence of stigma, impacting 74 (27.3%) of the participants (Fig 4) and (S4 Table).

**Fig 4:**
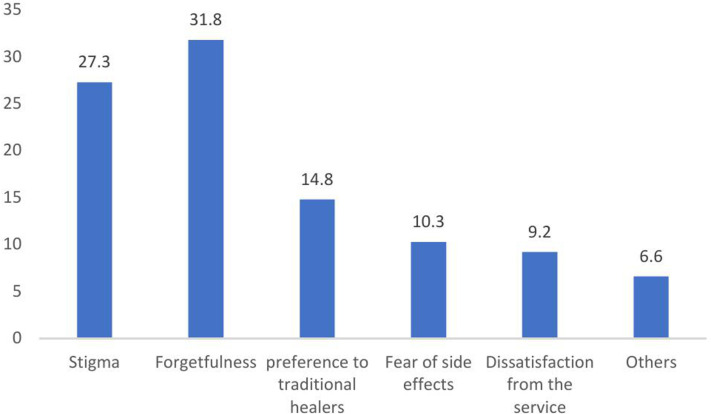
Reasons for non-adherence of patients with schizophrenia, Ethiopia (N **=** **271).** Others: lack of information, being busy, lack of efficacy, lack of insight.

### 3.4. Determinants of non-adherence to SGAs

The multivariate binary logistic regression analysis results demonstrated a significant association between non-adherence to SGAs and several key factors. A high PANSS score (p = 0.02), a CGI-S score of moderate to markedly severe illness (p = 0.024), a DUP (6-10) years (p = 0.02), and age (26-40) years (p = 0.04) were found to be statistically significant predictors of non-adherence to SGAs (Table 4).

**Table 4:** Multivariate binary logistic regression analysis of non-adherence of patients with schizophrenia, Ethiopia (N = 271).

Variables	COR (95% CI)	AOR (95% CI)
PANSS- mean & SD (71.1 ± 35.9)		
<71	1	1
>=71	1.9 (1.2,3.4)	1.8 (1.04, 3.80) *
CGI-S		
Borderline to mildly ill	1	1
Moderate to markedlysevere ill	1.7 (0.99,2.90)	1.9 (1.1,3.1) *
DUP		
<1yr	1	1
1-5yrs	0.9 (0.5,1.6)	0.9 (0.5, 1.8)
6-10yrs	11.4 (1.3,99.0)	13 (1.4, 129) *
>10yrs	2.3 (0.31,16.6)	2.8 (.34, 22.4)
Religion		
Orthodox Christian	1	1
Muslim	0.8 (0.50,1.50)	0.3 (.04, 2.0)
Protestant	0.6 (0.30,1.50)	0.2 (0.1, 1.7)
Others	3.0 (0.50,18.4)	0.2 (.02, 1.3)
Age category		
18-25	1	1
26-40	2 (0.7,6.7)	0.5 (0.3, 0.9) *
41-50	1.2 (0.5,3.5)	0.7 (0.3,1.6)
51-85	1.7 (0.5,5.6)	0.3 (0.1, 1.1)
Alcohol use		
Yes	1.6 (0.9,3.0)	1.7 (0.9,3.2)
No	1	1
Employment status		
Employed	1	1
Unemployed	1.4 (0.8,2.4)	1.5 (0.8, 2.5)

*P-value < 0.05.

AOR: Adjusted odds ratio; CGI-S: Clinical Global Impressions-severity Scale; COR: Crude odds ratio; DUP: Duration of untreated psychosis; PANSS: Positive and Negative Syndrome Scale; SD: Standard deviations

## 4. Discussion

This study investigates non–adherence and identifies predictors and reasons for medication non–adherence among Ethiopian patients with schizophrenia who are receiving SGAs treatment. The study revealed that approximately 31.4% of the participants were classified as non-adherent to their prescribed SGAs medication. This prevalence underscores that non–adherence is common among patients who are on SGAs.

Our study’s finding on medication non–adherence prevalence is lower than similar investigations conducted in various regions. For example, a report from Assela [13] showed non-adherence rate of 37.7% and another study in centeral Ethiopia found a rate of 41% [17]. A study conducted in India [41] showed a non-adherence rate of 52%. Other studies reported rates ranging 40-50% [18]. The differences in prevalence rates could be attributed to the inclusion of patients on both FGAs and SGA in their investigations. The presence of FGAS, known for their more pronounced side effects, could potentially contribute to lower adherence levels among patients in those studies [42,43]. Besides, in some studies the sample size was small which may result in an overestimation of the non-adherence levels. However, the finding in the present study is higher than what were reported in northern Ethiopia, Canada and USA [16,15,44]. This can be plausibly explained by several factors related to methodology, demographic differences, healthcare systems, different educational backgrounds, and cultural attitudes that affect their medication adherence behaviors [5,32,34].

The multifaceted nature of non–adherence to medication regimens among schizophrenia patients is evident in the diverse reasons reported across different studies and geographic regions. One of the reasons affecting non-adherence that emerged from our findings is the impact of forgetfulness on medication non–adherence. Forgetfulness was the most commonly cited reason for non–adherence, affecting nearly one-third of the participants (31.73%). Stigma was another critical factor contributing to non-adherence, reported by 27.31% of the participants. The stigma associated with mental illness can significantly deter patients from adhering to their prescribed medications. This finding aligns with a study conducted by Alene et al., which indicated forgetfulness (36.2%) as the typical reason for non-adherence to treatment [14]. Similarly, studies in Germany [45] and Canada [46] support these findings. One systematic review revealed that experiences of stigma and economic difficulties were the most common reason for non-adherence and highlighted recall problem as a common factor contributing to non–adherence, with reported proportions of 33.3% and 36.3%, respectively [47]. The prevalence of forgetfulness as a reason for non–adherence across diverse populations suggests a universal challenge that warrants targeted interventions [34,35].

Comparisons of studies conducted in different geographical regions revealed a spectrum of reasons for non–adherence, reflecting the complex interplay of cultural, social, and economic factors. For instance, in Gondar, Northwestern Ethiopia, recovery from the illness and seeking alternative treatment were reported as common reasons for non-adherence [15]. Likewise, studies in Butajira, central Ethiopia, identified inadequate availability of food to counter appetite stimulation and the perceived strength of antipsychotic medications as significant contributors to non–adherence [23]. In Turkey, denial was emerged as the leading reason for non–adherence [48], while in Pakistan, the choice of alternative treatment pathways such as traditional faith healers (96.4%) and lack of awareness (65.5%) were reported as the most common reasons for non–adherence [49]. Studies conducted in India highlighted denial of illness and financial burden as major reasons for non–adherence along with poor financial condition of the patients [20]. Getting better is also a common reason for treatment discontinuation in most cases of adherence studies in Southeast Asia [50]. A systematic review corroborated this diversity, emphasizing lack of illness insight, patients’ perception of medications effectiveness, and substance abuse as common factors influencing non–adherence [8]. In the USA, non-improvement of symptoms and medication-related adverse events were reported as prevalent reasons for non–adherence [51]. Lack of insight was viewed as the most important cause of medication discontinuation, followed by patients feeling better and thinking their medication was unnecessary and experiencing undesirable side effects in a survey conducted across Europe, the Middle East, and Africa [52]. The diverse reasons reported in different contexts underscore the importance of considering local beliefs, cultural nuances, and socioeconomic factors when designing interventions to improve medication adherence.

The multivariate binary logistic regression analysis provided insight into the predictors of non–adherence. Significantly, an elevated PANSS score, indicative of heightened symptom severity, was linked to non–adherence. In our study, patients with a PANSS score equal to or greater than 71 exhibited 1.8 times higher odds of non–adherence than those with a PANSS score below 71 (95% CI: 1.04, 3.80; p = 0.02). This finding is in line with the research conducted in Hong Kong [53] and South Korea [54] and India [20] where non–adherent patients demonstrated higher PANSS scores. A separate study in the United States aligns with our findings, indicating that higher PANSS positive factor emerged as the most robust predictor of treatment adherence, regardless of the specific medication used [55]. A comparable result was observed in China, where factors contributing to inadequate adherence behavior encompassed more pronounced positive symptoms [56]. This finding highlights the intricate relationship between symptom severity and medication non adherence. It suggests that improved symptom management may help enhance adherence rates. A rise in positive symptoms, including delusions, hallucinations, and aggressive behavior, might hinder adherence by fostering paranoia or uncooperativeness [5,34,43]. Similarly, an elevation in negative symptoms may compromise adherence, given the diminished volition or motivation to engage in the treatment process [57,58].

The presence of a moderate to markedly severe illness, as indicated by the CGI-S score, was also linked to non–adherence. Patients categorized as having a moderate to markedly severe illness exhibited 1.9 times higher odds of non-adherence compared to those classified as borderline to mildly ill, with a (95% CI: 1.1, 3.1; p = 0.024). This indicates that individuals with more severe illnesses may face additional challenges in adhering to their medication regimens. This finding aligns with research conducted in Hungary, where a notable inverse relationship was identified between compliance and scores on the CGI-S scale [59]. A study conducted in China [60,61] and France [62] indicated that increased disease severity, CGI-S scores of ≥ 4, is a predictive of non-adherence level.

Contrastingly, a study carried out in Canada did not reveal a correlation between disease severity and non-adherence levels [63]. This disparity could potentially be attributed to the utilization of a small sample size in that study, which might be leading to an underestimation of the association, or Patients with high disease severity may get better treatment follow-up which may increase adherence level [64,65].

Moreover, our study identified longer DUP as a predictor of non–adherence. The likelihood of non–adherence among patients with a DUP ranging from 6 to 10 years was 13 times higher than that of patients with a DUP of less than one year (95% CI: 1.4, 129; p = 0.02). This finding underscores the importance of early intervention and treatment initiation. Reducing the DUP has implications for symptom management and contributes to better adherence to medication regimens. Consistent with this, a study conducted in Egypt [66] and France [67] observed a parallel trend, noting that non–adherence rose in conjunction with the DUP. The mechanism linking extended DUP to non–adherence may be multifaceted. Prolonged exposure to psychotic symptoms can lead to greater impairment in cognitive functions and social functioning, making it more challenging for patients to adhere to treatment regimen [68]. In addition, another study reported that extended DUP was associated with a decrease in temporal and occipitotemporal gray matter volume in treatment-naïve schizophrenia patients, suggesting that brain structural changes due to untreated psychosis might contribute to poor treatment response and long-term prognosis [69].

Age, particularly within the age group of 26-40 years, emerged as a statistically significant predictor of non–adherence. The odds of non–adherence in this age group were reduced by 50% compared to the 18-25 age years group (95% CI: 0.3 to 0.9; p = 0.04). A plausible explanation for this finding could be that individuals in the age group of 26-40 years may assume greater responsibility for self-care than their younger counterparts. This finding aligns with research conducted in the United States indicating that adherence is less common among younger patients [70]. A comparable result was reported in China [53], wherein the group exhibiting non-adherence was notably younger in age.

On the contrary, a research finding from Mekelle showed that being older is associated with medication non–adherence [16]. This variation could be attributed to comorbidities and polypharmacy in those patients associated with increased risk of adverse drug reactions, which can further discourage patients from adhering to their medication regimens [8,71]. Differences in the approaches used to assess adherence levels could also play a role.

The present study comes with certain limitations. The determination of adherence levels relies on a self-reported measuring scale, contingent upon the honesty and trustworthiness of respondents. This dependence may introduce recall bias, potentially leading to overestimation of medication adherence. Additionally, the cross-sectional design cannot follow up with patients, precluding the establishment of causal relationships. Furthermore, the generalizability of findings may be constrained due to the single-centered nature of the study. In addition, the higher number of men compared to women in our study may pose limitation. This gender imbalance could affect the generalizability of our findings and may overlook gender-specific factors influencing medication adherence. Nonetheless, the study’s strengths, such as a robust sample size, randomized sampling techniques, and the utilization of both patient interviews and medical chart reviews, could help mitigate these limitations.

## 5. Conclusion

Our findings indicated that 31.4% of the participants were non–adherent to their prescribed SGAs medication. Investigating the reasons for non–adherence, forgetfulness was identified as the most commonly cited factor affecting 31.73% of the participants, followed by stigma, 27.31%. A high PANSS score, indicating greater symptom severity, a moderate to markedly severe illness, as indicated by the CGI-S score, and longer DUP and age were identified as statistically significant predictors of non-adherence. The investigators recommend counseling the patients to highlight the importance of adherence, instituting regular and comprehensive symptom monitoring, tailoring interventions to address reasons for non-adherence, promoting early intervention and treatment initiation to reduce the DUP, and customizing interventions based on age-specific needs.

## Supporting information

S1 Table Adherence status with respect to years of untreated psychosis.
(PDF)

S2 Table Adherence status with respect to CGI-S score.
(PDF)

S3 Table Adherence status with respect to PANSS score.
(PDF)

S4 Table Reasons for non-adherence.
(PDF)
