# Supplementary material for: Non–adherence and predictors in patients with schizophrenia on second generation antipsychotics at Amanuel Mental Specialized Hospital, Ethiopia
Source: PLoS One. 2025 Mar 26;20(3):e0314403. doi: 10.1371/journal.pone.0314403 (PMC11940446; doi:10.1371/journal.pone.0314403)
Supplement: S1 Table — (PDF) [file pone.0314403.s001.pdf]

S1 Table. Adherence status with respect to years of untreated psychosis.

		duration of untreated psychosis				
		<1 year, N(%)	1-5 yrs, N(%)	6-10 yrs, N(%)	>10 yrs, N(%)	Total, N(%)
Adherence status	adherent	132(69.5)	51(71.8)	1(16.7)	2(50.0)	186(68.6)
	non adherent	58(30.5)	20(28.2)	5(83.3)	2(50.0)	85(31.4)
Total		190(100)	71(100	6(100)	4(100)	271(100)
