# Supplementary material for: Non–adherence and predictors in patients with schizophrenia on second generation antipsychotics at Amanuel Mental Specialized Hospital, Ethiopia
Source: PLoS One. 2025 Mar 26;20(3):e0314403. doi: 10.1371/journal.pone.0314403 (PMC11940446; doi:10.1371/journal.pone.0314403)
Supplement: S2 Table — (PDF) [file pone.0314403.s002.pdf]

**S2 Table. Adherence status with respect to CGI-S score.**

| | | CGI-S | | |
|---|---|---|---|---|
| | | Borderline to mildly ill, N(%) | moderate to markdly sever ill, N(%) | Total, N(%) |
| Adherence status | adherent | 117(74.1) | 69(61.1) | 186(68.6) |
| | non adherent | 41(25.9) | 44(38.9) | 85(31.4) |
| Total | | 158(100) | 113(100) | 271(100) |
