# Supplementary material for: Non–adherence and predictors in patients with schizophrenia on second generation antipsychotics at Amanuel Mental Specialized Hospital, Ethiopia
Source: PLoS One. 2025 Mar 26;20(3):e0314403. doi: 10.1371/journal.pone.0314403 (PMC11940446; doi:10.1371/journal.pone.0314403)
Supplement: S4 Table — (PDF) [file pone.0314403.s004.pdf]

S4 Table. Reasons for non adherence.

	Frequency	Percent
Stigma	74	27.3
Forgetfulness	86	31.7
Preference to traditional healers	40	14.8
Fear of side effects	28	10.3
Dissatisfaction from the service	25	9.2
Others*	18	6.6
Total	271	100.0

* lack of information, being busy, lack of efficacy, lack of insight
